# Assessing the Relationship between Body Image Satisfaction and Physical Activity in Italian Adolescents: A Cross-Sectional Investigation

**DOI:** 10.3390/children11070818

**Published:** 2024-07-03

**Authors:** Sabrina Masotti, Tommaso Piva, Valentina Zerbini, Andrea Raisi, Erica Menegatti, Anselmo Pagani, Costanza Bigoni, Elena Ballarin, Federica De Luca, Luciana Zaccagni, Natascia Rinaldo, Stefania Toselli, Emanuela Gualdi-Russo, Antonio Argentoni, Arli Veli, Gianni Mazzoni, Simona Mandini

**Affiliations:** 1 Center for Exercise Science and Sport, Department of Neuroscience and Rehabilitation, Faculty of Medicine, Pharmacy and Prevention, University of Ferrara, 44121 Ferrara, Italytommaso.piva@unife.it (T.P.); andrea.raisi@unife.it (A.R.); luciana.zaccagni@unife.it (L.Z.); natascia.rinaldo@unife.it (N.R.); emanuela.gualdi@unife.it (E.G.-R.); antonio.argentoni@unife.it (A.A.); gianni.mazzoni@unife.it (G.M.); simona.mandini@unife.it (S.M.); 2 PhD Program in Environmental Sustainability and Wellbeing, University of Ferrara, 44121 Ferrara, Italy; 3 Department of Environmental and Prevention Sciences, University of Ferrara, 44123 Ferrara, Italy; 4 PhD Program in Translational Neurosciences and Neurotechnologies, University of Ferrara, 44121 Ferrara, Italy; 5 Filippo De Pisis Lower Secondary School, 44124 Ferrara, Italy; 6 Department of Life Quality Studies, University of Bologna, 47921 Rimini, Italy; stefania.toselli@unibo.it

**Keywords:** body image, anthropometry, physical activity, body satisfaction, body dissatisfaction

## Abstract

Background: Body image perception can significantly influence various aspects of adolescent lives. The study analyzed the relationship between body image satisfaction and sports participation in adolescents, examining various factors that contribute to body image concerns and their implications for sports engagement. Methods: A total of 237 schoolchildren were recruited from lower secondary Italian schools. Anthropometric characteristics were measured directly. Assessment of body image perception was performed using Body Silhouette Charts for preadolescent children. The Italian version of the International Physical Activity Questionnaire for Adolescents questionnaire was administered to assess physical activity (PA) levels at school and during leisure time in the last 7 days. Analysis of variance was used to test differences in PA levels, while multiple regression models were carried out to assess possible predictors of body dissatisfaction. Results: In total, 42.6% of children were not satisfied with their figure and 23.2% were very dissatisfied; among them, the vast majority would have liked to be thinner. The dissatisfaction and satisfaction were similar in boys and girls. The frequency of satisfaction with one’s body image was higher in children who practiced extracurricular sports compared to those who did not. The percentage of dissatisfaction with one’s body image was similar in the two groups, but the frequency of children being very dissatisfied was double in the group that does not practice extracurricular sport (31.2% vs. 17.7%). Body dissatisfaction increases with increasing body mass index in both genders but decreases in children involved in extracurricular sports. Conclusions: Encouraging teenagers to engage in regular physical activity should be a key component of therapies supporting positive body image. This study found a relationship between extracurricular sports and body image satisfaction, suggesting that physical activity protects teenagers’ body image satisfaction.

## 1. Introduction

Adolescence is a crucial developmental stage characterized by numerous physical, emotional, and social changes. During this period, adolescents often experience heightened self-awareness and self-consciousness, including concerns about their physical appearance [1,2]. Body image perception, defined as individuals’ subjective evaluation of their own body’s size, shape, and attractiveness, becomes particularly salient during adolescence and can significantly influence various aspects of their lives [3].

Physical activity participation holds great potential as a context that can impact body image perception in adolescents. Engaging in sports not only offers opportunities for physical fitness but also provides an environment where individuals can develop skills, interact with peers, and establish a sense of belonging [4]. Moreover, participation in sports can foster positive body image through the cultivation of physical competence, improved self-esteem, and appreciation for diverse body types [5]. As regards physical activity, it decreases throughout adolescence; this pattern may translate into lower physical activity rates throughout the lifespan. Given the numerous physical, emotional, social, and spiritual health and well-being advantages connected with physical activity, the tapering of engagement throughout early adolescence warrants more investigation.

However, body image concerns can also manifest in the context of sports participation, as athletes may face heightened pressures and expectations related to their physical appearance and performance [6]. Competitive sports environments, media portrayal of idealized bodies, and comparisons with peers can contribute to negative body image perceptions, leading to psychological distress, disordered eating behaviors, and a reduced willingness to participate in physical activities [7].

Understanding the complex relationship between body image perception and sports participation in adolescents is essential for designing effective interventions and promoting positive youth development [8,9]. Sometimes, body image perception may induce motivation or dissuasion for PA and sports participation. Misperception of body image can lead to exercise addiction and can result in decreased performance owing to overload and physical burnout [10]. By examining the multifaceted factors influencing body image perceptions within the sports context, researchers and practitioners should identify strategies to optimize the benefits of sports participation while minimizing the potential negative consequences on body image.

The definition of body image that is most frequently employed is a multidimensional construct that emphasizes both the look and function of the body. The importance of body image in relation to physical exercise and sporting behavior has drawn a lot of attention. In terms of sport and exercise psychology, body image is important as a correlate, antecedent, and result of physical activity (such as structured exercise, leisure, and lifestyle physical activity) and sport (such as competitive and/or recreational activity requiring skill and physical exertion) behavior. Multiple aspects of one’s body image may be involved in the engagement of physical activity and sports behavior [7].

Several cross-sectional studies have linked different body-related self-conscious attitudes to physical activity (PA) behavior. Body-related shame has been consistently associated with reduced PA levels, whereas guilt has been associated with inconsistent PA behavior [7,9]. Despite the importance of this topic, studies involving sport-related body image in adolescents are scarce. The current study could also have a possible contribution to the existing literature [11,12,13].

This paper aims to explore the relationship between body image satisfaction and sports participation in adolescents, examining various factors that contribute to body image concerns and their implications for sports engagement. Additionally, we will highlight gaps in current knowledge, propose potential avenues for future research, and discuss practical implications for promoting positive body image and enhancing sports experiences among adolescents.

## 2. Methods

### 2.1. Study Design and Participants

The present cross-sectional and observational study consists of a total of 237 schoolchildren, 144 boys (60.8%) and 93 girls (39.2%) aged between 12 and 15 (mean: 13.1 ± 0.8 years in the whole sample; 13.2 ± 0.7 years in males; 12.9 ± 1.1 years in females) recruited from lower secondary schools in Ferrara (northern Italy). Written assent from all participants and consent from one parent or legal tutor was obtained. Before administering the questionnaire, the researcher briefly explained the procedure and gave instructions on completing the questionnaires. The exclusion criteria were not having parental written consent and not completing all the parts of the questionnaires. The study protocol was approved by a local Bioethics Committee of the University (Bologna; Ethical Approval no. 2.18).

### 2.2. Anthropometry

Anthropometric traits were directly measured by one researcher according to standardized procedures [11,14]. Body weight was measured using a mechanical scale (precision 0.1 kg; SECA GmbH & Co. KG., Hamburg, Germany), and standing height was determined with a portable stadiometer (precision 0.1 cm; Magnimeter, Raven Equipment Limited, Dunmow, Essex, UK). Waist circumference (WC) was measured using a non-stretchable tape (precision 0.1 cm; DKSH, Zurich, Switzerland). Body mass index (BMI) was calculated as weight (kg)/height (m^2^) and the nutritional status of children was categorized as underweight, normal weight, overweight, and obese according to international age and sex-specific Cole’s cut-off points (Table 1) [15,16].

### 2.3. Body Image

Assessment of body image perception was performed using Body Silhouette Charts for preadolescent children [17]. The figure rating scale consists of eight figures, drawings for each gender, and representing several body outlines, ranging from very slim (silhouette 1) to obese (silhouette 8) [17]. Participants were asked to choose the silhouette best representing how they currently look (Feel) and the silhouette showing how they ideally wanted to look (Ideal). Participants could not choose an intermediate value between those of two successive silhouettes.

The degree of body dissatisfaction was assessed through the Feel minus Ideal Discrepancy (FID) index defined as the difference between the perceived current figure (Feel) and the Ideal figure [18]. A positive FID score indicates the desire to be thinner and a negative FID score indicates the desire to be fatter. An FID score of 0 indicates no discrepancy (same figure chosen as Feel and Ideal). Three dummy variables were created for body image satisfaction according to Mendo-Lazaro et al. [1], based on the difference between the Feel figure and Ideal figure: satisfied (Feel = Ideal), dissatisfied (Feel-Ideal = ±1), and very dissatisfied (Feel-Ideal ≥ +2 or −2).

### 2.4. Physical Activity

The Italian version of the International Physical Activity Questionnaire for Adolescents questionnaire (IPAQ-A) was administered to assess PA levels at school and during leisure time in the last 7 days [19].

The questionnaire includes questions regarding four domains (school, transportation, household, and leisure time), for which total minutes per week were computed, followed by minutes per week for walking, moderate, and vigorous PA based on IPAQ data processing and analysis criteria. The Metabolic Equivalent of Task (MET) score was computed for the week in three different physical activity intensities: walking (METWalkwk), moderate (METModeratewk), and vigorous (METVigorouswk) and for overall estimated total MET per week (METwk).

According to the authors that validated this procedure, it should be self-administered but providing support is allowed especially for younger students.

### 2.5. Other Background Data

General information about demographics (e.g., gender and age), extracurricular sports, and the amount of time involved (hours per week) were collected through a questionnaire. Sports were categorized into two typologies: team sports and individual sports. These sports variables were measured dichotomously (yes/no).

### 2.6. Statistical Analysis

Descriptive statistics information was presented as means and standard deviation (SD) or frequencies (%) for the total sample and grouped by gender. Distribution normality was assessed using the Kolmogorov–Smirnov test. Gender comparisons for categorized variables were investigated through the chi-square test, while differences in the means of the continuous variables were tested using the Student’s *t*-test or Mann–Whitney test when the examined traits were found to have a normal or non-normal distribution, respectively.

Pearson’s correlation was carried out to determine the associations between variables and classified as small (0.10–0.29), medium (0.30–0.49), and large (>0.50) [20].

ANOVA was used to test the differences in the mean of METwk score in relation to three dummy variables of satisfaction.

Backward multiple regression analysis was carried out to assess possible predictors of FID. Anthropometric characteristics (BMI and WC), extracurricular sports practice (yes/no), and different PA intensities (met/week) were included in the regression models as independent variables. Extracurricular sports practice was included in the model as a binary variable (extracurricular sports practice = 1; no extracurricular sports practice = 0). Multicollinearity among the variables was examined by variance inflation factors, assuming values between 0.10 and 10 as acceptable variance inflation factor.

All analyses were performed using the statistical program “Statistica” for Windows, Version 12.0 (StatSoft Srl, Tulsa, OK, USA), and the significance level was set at 5% (*p* < 0.05).

## 3. Results

Anthropometric characteristics, body image perception and satisfaction, PA, and sports practice in the total sample and relation to gender and sports practice are reported in Table 2.

### 3.1. Modulating Variables by Gender

The majority of the total sample had a normal weight; however, almost a third of the sample was overweight or obese. The frequency of overweight was slightly higher in males than females, while the percentage of obesity was similar in both genders.

Analyses revealed statistically significant differences between males and females for the following variables: waist circumference, Feel and Ideal figure, METVigorouswk, METwk, extracurricular sports practice (Yes/No), and type of sports (individual/team sport) (Table 2).

More specifically males showed higher WC values from an anthropometric point of view. As regards the other variables, males had higher values in the choice of Feel and Ideal figures, as well as MET consumption for vigorous PA (METVigorouswk), and total weekly MET consumption (METwk).

There were no significant differences in body image satisfaction between genders.

The FID score showed positive discrepancies, indicating a misperception tending to overestimation in both genders.

In total, 67.4% of boys practiced extracurricular sports compared to 44% of girls. Regarding the type of sport, an inverse trend is observed between the two genders where males play more team sports (74.2%) while females play more individual sports (61.4%).

Concerning the body silhouette figures, both genders seemed more likely to select F.4 to represent their body image (Table 3), while the figures more representative of their ideal body image were F.4 and F.3 in boys and girls, respectively. The children showed a preference for thinner bodies since both genders had significantly (*p* < 0.0000) lower mean values of the Ideal figures (boys: 3.85 ± 0.90; girls: 3.21 ± 0.79) than of the Feel figures (boys: 4.56 ± 1.18; girls: 4.04 ± 1.23). However, there was a positive correlation between the Feel figure and the Ideal figure in both genders (boys: r = 0.3302, *p* < 0.0000; girls: r = 0.3132, *p* < 0.0022).

Table 3 reports an increase in mean BMI values with increasing values of the silhouette in both genders. Moreover, Feel figure perception and BMI were significantly and positively correlated in both male and female adolescents (boys: r = 0.7263 *p* < 0.0000; girls: r = 0.6259 *p* < 0.0000). These results provide a measure of the general appropriateness of the children’s body image perception. The exceptions were two girls who showed a misperception of their body image by choosing the Feel figure F7 in the first case and F8 in the second case, despite having a BMI equal to 29.0 and 26.4, respectively.

In total, 42.6% of children were not satisfied with their figure (FID = ±1) and 23.2% were very dissatisfied (FID ≥ ±2) (Table 2); among them, the vast majority would have liked to be thinner. The dissatisfaction and satisfaction were similar in boys and girls (Table 2). The FID values indicated slightly greater dissatisfaction in the girls than in the boys. The frequency of dissatisfied adolescents increased significantly in overweight and obese in both genders (Table 4). There were no statistically significant differences in the score of METwk about body satisfaction in both genders, although there was a trend towards a decrease in the girls being very dissatisfied (Table 4).

It was also noted that regarding body image satisfaction there were no statistically significant differences between children who practiced extracurricular sports and those who did not practice them in both genders (Table 4); however, the total sample showed statistically significant differences between the two groups (Table 2).

### 3.2. Modulating Variables by Extracurricular Sport Practice

The total participants were divided into two groups based on whether or not they practice extracurricular sports (Table 2). On the basis of this comparison, statistically significant differences were observed between the two groups in the BMI value, in the choice of the Feel and Ideal figure, and the FID score. All values are higher in the group that did not practice extracurricular sports compared to the one that practiced extracurricular sports. Body image satisfaction showed statistically significant differences between the two groups (Table 2). The frequency of satisfaction with one’s body image was higher in children who practiced extracurricular sports compared to those who did not participate in extracurricular sports. The percentage of dissatisfaction with one’s body image was similar in the two groups, but the frequency of children being very dissatisfied was double in the group that does not practice extracurricular sport (31.2% vs 17.7%) (Table 2). There are also statistically significant differences between the two groups in consumption of Met in vigorous PA (METVigorouswk) and per week (METwk).

Table 5 shows the multiple linear regression analysis results for the total sample, conducted with all the variables input into the model with backward methods. The analysis was used to investigate whether the FID score of the subjects could be explained by their anthropometric characteristics (BMI and WC), age, extracurricular sport practice (yes/no), and different PA intensities (METWalkwk; METModeratewk; METVigorouswk and METwk). In the multivariate model, BMI and WC were significant predictors positively associated with FID, while extracurricular sport practice was negatively associated. This model accounted for 41.0% of the variance of FID in children.

## 4. Discussion

The present study aimed to evaluate the relationship between body image satisfaction and sports participation associated with weight status and gender in a sample of Italian adolescent students. Additionally, this study sought to determine if variations in body image satisfaction were brought about by extracurricular PA or influenced by the child’s gender, PA level, or all these variables.

The perception of our body is a dynamic characteristic that can change throughout life. Because of the age-related changes that occur throughout this stage of life, adolescence is a critical period for the formation of a positive body image. Studies have shown that there are gender differences when it comes to general self-conscious emotions like guilt, shame, and humiliation. Typically, males tend to experience these emotions less than females, while there are no gender differences in degrees of genuine and hubristic pride.

The majority of the participants in this research were of normal weight, but there was still an alarming number of overweight and obese children. The findings of this study show that there were no gender differences in frequencies of categories of nutritional status. The lack of disparities in weight status is consistent with a recent Italian study [21] and disagrees with some studies that reported a significantly higher proportion of overweight-obese males compared with females [22,23]. In our research, the frequencies of overweight and obese children were higher than in the national data for both genders [24]; in particular, the percentage of obese girls was higher than the national trend [24].

Obesity has increased in children and young people since 1975 all over the world but especially in developed countries [25]. In the European region, the highest levels of overweight and obesity are reported in Mediterranean and Eastern European countries [26]. Childhood obesity can lead to a variety of serious complications in adulthood such as metabolic and cardiovascular diseases and psychosocial consequences such as a poor opinion of one’s body image and low self-esteem. Low levels of PA are one of the causes of obesity in developed countries, in particular, obese adolescents are generally less active than those of normal weight [26,27]. In prepubertal and pubertal children, PA can lead to a reduction in visceral and trunk fat.

Concerning body image and BMI values, the results suggested that the perception of one’s body image tended to be adequate in the total sample. The assessment of the distortion of body image is an important tool for preventing the onset of eating disorders and identifying cases that necessitate intervention during growth. The strong relationship between anthropometric variables and body image perception plays a fundamental role in nutritional surveillance programs.

In this study the analysis of the ideal silhouette showed a thinner ideal body image in both genders. Moreover, the FID was higher in females than in males, although there were no statistically significant differences between the genders. In the literature it is reported that girls are more sensitive and more dissatisfied with their bodies than boys [9,28,29,30], although in recent years the role of gender has changed and the continuous exposure of thin bodies by the mass media affects the perception of body image of both genders. Media pressure regarding beauty standards can lead to the adoption of negative behaviors for weight reduction such as restrictive diets which can lead to eating and mental disorders [31,32]. Our results show similar percentages of girls and boys satisfied, dissatisfied, and very dissatisfied with their bodies. We found 83.3% of the dissatisfied sample would like to be thinner. Vaquero et al. [33] found that 50% of children between 7 and 12 years of age would like to be slimmer. Our research seems to agree with some studies [1,34,35,36] that report dissatisfaction with one’s body image in both genders equally.

The level of dissatisfaction increased with increasing weight status in both genders. Through multiple regression analysis, a clear relationship between FID and BMI was found: BMI is significantly and positively correlated with body image dissatisfaction. WC also appears to be an anthropometric variable strongly correlated to FID in terms of body shape that can influence body image perception [37]. The subjects in this study were in an evolutionary period in which the shape of their body underwent considerable changes. These changes can affect body image perception positively or negatively. This result agreed with other studies [6,23], which found that increased BMI influences body image perception. Pinho et al. [28] report a high dissatisfaction in particular in overweight adolescents.

The practice of extracurricular sports did not show significant associations with body image dissatisfaction in both genders; however, the influence of extracurricular sports on body dissatisfaction is consistent in the total sample. This could be due to the low sample size of both genders participating in extracurricular sports. The analysis showed that the frequency of children with high dissatisfaction with their body image was higher in those who did not practice extracurricular sports. In this case, the FID score was double that of those who practice extracurricular sports. Our regression analysis showed that FID was associated with extracurricular sports practice in the total sample. This, in agreement with other studies [38,39], suggested the existence of a relationship between the practice of extracurricular sports and the satisfaction of one’s body image. Several studies [40,41,42] indicate that children who practice PA have a better perception of their body image. This means that the intensity, frequency, competitiveness, and commitment to sport strongly affect the emotional sphere of the subjects [43]. The benefits of PA in adolescents are seen in health status, improved fitness and body image, and general enjoyment [44]. Brambilla et al. [45] found that PA is perceived as pleasant in children with obesity but participation in PA is shown to decrease in girls with poor body image when their weight is criticized.

This study found that there was no association between the score of METweek and body image satisfaction. Also, vigorous PA was not significantly associated with body satisfaction by gender and in the total sample, although male students spent more time in vigorous PA than female students. Several studies suggested that PA can reduce dissatisfaction with body image [9]. However, some studies showed other influencing factors in the relationship between PA and body image satisfaction including the individual’s motivation for exercising, internalization of ideal body size, and environmental factors [46]. Furthermore, exercise behavior can sometimes increase body image dissatisfaction [47]. For all these reasons, it is evident that the relationship between PA and body image satisfaction was complex and indicates a need for further investigation. Our research showed higher METvigouroswk in girls who were satisfied with their body image, although these data were not statistically significant. Fisher et al. [48] found that vigorous-intensity PA was positively associated with happiness among girls but not boys. This difference could be due to biological factors such as dopamine, which has different regulation mechanisms about sex [49,50]. Labbrozzi et al. [51] also found that younger girls were more motivated towards PA practice, reporting more enjoyment. Fun and motivation were reduced in the following growth phases showing a worsening of the perception of one’s body image in girls.

Our findings were consistent with a prior study that suggested PA protects teenagers’ body image satisfaction [3,52].

### Strength and Limitations

The strength of this study was the direct measurement of anthropometric traits of the children by anthropometric experts, not self-reporting.

However, we observed the following limits: (a) the sample size split into groups by sport practice was limited, and, in the future, it will be necessary to increase the sample to verify the validity of the present results; (b) this was a cross-sectional study; a longitudinal study throughout the adolescence period would be necessary to verify any changes in the relationships of the parameters considered; (c) the research took place in the post-COVID 19 period and could be influenced by behaviors undertaken during the restrictions of the pandemic, such as a reduction in PA and an increase in sedentary lifestyle [53].

## 5. Conclusions

This study provides new data on body image perception in Italian adolescents, contributing to the knowledge of body image satisfaction in boys and girls about weight status and PA. In the present research, BMI, WC, and extracurricular sports practice were the main determinants of body image satisfaction. The results confirm the great dissatisfaction of overweight or obese participants. Body dissatisfaction increases with increasing BMI in both genders but decreases in children involved in extracurricular sports. Therefore, encouraging teenagers to engage in regular PA should be a key component of therapies that support positive body image and guard against nutritional problems.

## Figures and Tables

**Table 1:** Cut-off points for body mass index for underweight, overweight, and obesity by sex and age applied to sample [12,13].

Age (Years)	Underweight		Overweight		Obesity	
	Males	Females	Males	Females	Males	Females
12.0	15.35	15.62	21.22	21.68	26.02	26.67
12.5	15.58	15.93	21.56	2214	26.43	27.24
13.0	15.84	16.26	21.91	22.58	26.84	27.76
13.5	16.12	16.57	22.27	22.98	27.25	28.20
14.0	16.41	16.88	22.62	23.34	27.63	28.57
14.5	16.69	17.18	22.96	23.66	27.98	28.87
15.0	16.98	17.45	23.29	23.94	28.30	29.11
15.5	17.26	17.69	23.60	24.17	28.60	29.29

**Table 2:** Characteristics of participants by gender, extracurricular sport practice group, and total sample.

Variable	Total Sample	Boys	Girls	*p*-Value	Extracurricular Sport Practice	No Extracurricular Sport Practice	*p*-Value
	Mean (SD)/% (N)	Mean (SD)/% (N)	Mean (SD)/% (N)		Mean (SD)/% (N)	Mean (SD)/% (N)	
**Anthropometric Measures**							
Height (cm)	156.19 (9.27)	156.78 (10.83)	155.26 (6.09)	0.2167	156.26 (9.79)	156.07 (8.51)	0.8731
Weight (kg)	50.60 (12.5)	51.10 (13.13)	49.83 (11.37)	0.4425	49.79 (12.26)	51.80 (12.72)	0.2225
WC (cm)	69.35 (9.75)	70.96 (10.16)	66.86 (8.55)	0.0014	68.80 (9.12)	70.16 (10.62)	0.2939
BMI (kg/m^2^)	20.54 (3.75)	20.54 (3.66)	20.55 (3.91)	0.9748	20.14 (3.34)	21.13 (4.23)	0.0448
**Weight Status**							
Underweight	8.9 (21)	8.3 (12)	9.7 (9)	0.8876	9.9 (14)	7.2 (7)	0.1822
Normalweight	63.7 (151)	62.5 (90)	65.6 (61)		66.7 (94)	59.4 (57)	
Overweight	21.9 (52)	23.6 (34)	19.3 (18)		19.8 (28)	25.0 (24)	
Obese	5.5 (13)	5.6 (8)	5.4 (5)		3.6 (5)	8.4 (8)	
**Body Image Perception**							
Feel figure	4.36 (1.23)	4.56 (1.18)	4.04 (1.23)	0.0013	4.21 (1.12)	4.57 (1.34)	0.0260
Ideal figure	3.60 (0.91)	3.85 (0.90)	3.21 (0.79)	0.0000	3.65 (0.85)	3.52 (0.99)	0.2770
FID (score)	0.76 (1.23)	0.71 (1.23)	0.83 (1.24)	0.4923	0.56 (0.99)	1.05 (1.47)	0.0023
**Body Image Satisfaction**							
Satisfied	34.2 (81)	33.3 (48)	35.5 (33)	0.8375	39.0 (55)	27.1 (26)	
Dissatisfied	42.6 (101)	42.4 (61)	43 (40)		43.3 (61)	41.7 (40)	
Very dissatisfied	23.2 (55)	24.3 (35)	21.5 (20)		17.7 (25)	31.2 (30)	0.0247
**Physical Activity**							
METWalkwk	900.83 (864.78)	952.50 (959.89)	820.52 (688.89)	0.2543	905.50 (880.52)	893.95 (845.64)	0.9202
METModeratewk	1072.0 (1350.48)	1123.89 (1479.74)	991.30 (1123.45)	0.4637	1164.29 (1482.43)	935.96 (1122.08)	0.2040
METVigorouswk	2084.25 (1562.71)	2335.38 (1636.15)	1693.91 (1359.85)	0.0019	2804.0 (1547.16)	1023.58 (794.37)	0.0000
METwk	4057.07 (2308.71)	4411.77 (2337.90)	3505.73 (2161.48)	0.0031	4873.78 (2307.13)	2853.49 (1714.68)	0.0000
**Extracurricular Sport Practice**							
Yes	59.5 (141)	67.4 (97)	47.3 (44)	0.0021	-	-	
Team sport	63.1 (89)	74.2 (72)	38.6 (17)	0.0000	-	-	
Individual sport	36.9 (52)	25.8 (25)	61.4 (27)		-	-	

**Table 3:** Frequencies of Feel and Ideal figures selected (18) and mean BMI of children choosing each Feel figure.

Silhouette Number	Boys			Girls		
	Feel Figure	Ideal Figure	BMI	Feel Figure	Ideal Figure	BMI
	% (N)	% (N)	Mean (SD)	% (N)	% (N)	Mean (SD)
F.1	0.0 (0)	2.8 (4)	-	1.1 (1)	1.1 (1)	15.68 (-)
F.2	1.4 (2)	4.2 (6)	14.68 (0.09)	6.4 (6)	16.1 (15)	16.20 (1.66)
F.3	17.4 (25)	18.7 (27)	17.72 (1.88)	25.8 (24)	46.2 (43)	19.34 (3.17)
F.4	34.0 (49)	55.5 (80)	18.71 (2.41)	37.6 (35)	33.3 (31)	19.55 (2.38)
F.5	24.3 (35)	17.4 (25)	21.65 (2.70)	15.0 (14)	3.2 (3)	22.62 (3.37)
F.6	16.7 (24)	1.4 (2)	23.95 (2.60)	11.8 (11)	0.0 (0)	25.26 (4.49)
F.7	6.2 (9)	0.0 (0)	26.17 (3.64)	1.1 (1)	0.0 (0)	29.05 (-)
F.8	0.0 (0)	0.0 (0)	-	1.1 (1)	0.0 (0)	26.43 (-)
Total	100 (144)	100 (144)		100 (93)	100 (93)	

**Table 4:** Body image satisfaction by weight status and physical activity.

	Body Image Satisfaction			*p*-Value
	Satisfied	Dissatisfied	Very Dissatisfied	
	Mean (SD)/N (%)	Mean (SD)/N (%)	Mean (SD)/N (%)	
**Boys**				
Weight status				
Underweight	6 (50.0)	3 (25.0)	3 (25.0)	0.0000
Normalweight	38 (42.2)	41 (45.5)	11 (12.3)	
Overweight	3 (8.8)	16 (47.0)	15 (44.2)	
Obese	1 (12.5)	1 (12.5)	6 (75.0)	
Physical activity				
METWk	4282.76 (339.45)	4415.87 (303.61)	4581.69 (397.52)	0.8491
METVigorouswk	2258.33 (237.70)	2366.0 (212.60)	2388.57 (278.37)	0.9220
Extracurricular sport practice				
Yes	36 (37.1)	41 (42.3)	20 (20.6)	0.1635
No	12 (25.5)	20 (42.5)	15 (32)	
**Girls**				
Weight status				
Underweight	3 (33.3)	6 (66.7)	0 (0.0)	0.0107
Normalweight	28 (45.9)	24 (39.3)	9 (14.8)	
Overweight	2 (11.1)	8 (44.4)	8 (44.5)	
Obese	0 (0.0)	2 (40.0)	3 (60.0)	
Physical activity				
METWk	4002.62 (374.43)	3305.0 (344.42)	3077.30 (481.0)	0.2407
METVigorouswk	1899.39 (236.0)	1727.18 (217.09)	1290.0 (303.14)	0.2835
Extracurricular sport practice				
Yes	19 (43.2)	20 (45.4)	5 (11.4)	0.0674
No	14 (28.6)	20 (40.8)	15 (30.6)	
**Total sample**				
Weight status				
Underweight	9 (42.8)	9 (42.8)	3 (14.4)	0.0000
Normalweight	66 (43.7)	65 (43.0)	20 (13.3)	
Overweight	5 (9.6)	24 (46.1)	23 (44.3)	
Obese	1 (7.7)	3 (23.1)	9 (69.2)	
Physical activity				
METWk	4168.63 (257.45)	3978.25 (232.88)	4034.64 (312.44)	0.8575
METVigorouswk	2112.10 (174.28)	2114.34 (157.64)	1989.09 (211.50)	0.8762

**Table 5:** Predictors of FID: multiple linear regression with backward method.

Predictor	β	t	*p*	VIF
BMI	0.3650	3.1205	0.0020	5.4318
Extracurricular sport practice (yes)	−0.1265	−2.4822	0.0137	1.0323
WC	0.2704	2.3273	0.0208	5.3595
R^2^	0.4180			
R^2^ adjusted	0.4105			
*p*	0.0000			

## Data Availability

The data that support the findings of this study are available on request from the corresponding author, V.Z. due to privacy reasons.
